# Platelet activation in adult HIV-infected patients on antiretroviral therapy: a systematic review and meta-analysis

**DOI:** 10.1186/s12916-020-01801-9

**Published:** 2020-11-18

**Authors:** Bongani B. Nkambule, Vuyolwethu Mxinwa, Zibusiso Mkandla, Tinashe Mutize, Kabelo Mokgalaboni, Tawanda M. Nyambuya, Phiwayinkosi V. Dludla

**Affiliations:** 1 School of Laboratory Medicine and Medical Sciences (SLMMS), College of Health Sciences, University of KwaZulu-Natal, Private Bag X54001, Durban, 4000 South Africa; 2 Biomedical Research and Innovation Platform (BRIP), Medical Research Council (MRC), Tygerberg, Cape Town, South Africa

**Keywords:** Platelets, P-selectin, Thrombosis, Cardiovascular disease, Antiretroviral therapy, HIV

## Abstract

**Background:**

Antiretroviral therapy (ART) alters platelet reactivity, and as a consequence, patients living with HIV may be at an increased risk of cardiovascular disease (CVD). The current evidence on platelet activation levels in patients with HIV remains inconclusive. We therefore aimed to systematically synthesise evidence on the association of platelet activation in HIV-infected patients on successful treatment.

**Methods:**

Electronic databases were searched from inception until November 2019. Studies were included if the primary or secondary outcome of the study was to assess platelet activation in HIV-infected patients on ART. The primary outcome of this review included the levels of platelet activation. The pooled effect estimates were calculated using a random-effects meta-analysis model.

**Results:**

We identified 30 studies comprising of 2325 participants. The pooled estimates showed elevated levels of platelet activation in treatment-naïve HIV-infected patients compared to uninfected controls (Hedges’ *g* 2.00 [95%CI 1.05, 2.94]; *z* = 4.12, *p* < 0.0001). These remained elevated despite successful ART (Hedges’ *g* 2.05 [95%CI 0.58, 3.52]; *z* = 2.71, *p* = 0.0067).

**Conclusion:**

The levels of platelet activation are elevated in treatment-naïve HIV-infected patients, and these persist during successful ART. Further studies should assess the clinical relevance of monitoring the levels of platelet activation in HIV-infected patients on ART.

## Background

In the era of combination antiretroviral therapy (cART), the overall life span of people living with HIV (PLWHIV) on ART is now almost similar to that of uninfected individuals [1, 2]. However, disparities in treatment outcomes still exist in low-income and high-income countries [3]. Amongst ART-treated patients, the most prevalent comorbidities include cardiovascular disease (CVD) and hepatic disorders [4]. CVD remain a challenge in the management of PLWHIV, with a persistent gap in the reported survival outcomes. The paucity of clinical trials focusing on cardiovascular outcomes of patients on long-term ART limits the availability of evidence-based patient management strategies. In a previous meta-analysis of cardiac dysfunction in PLWHIV, left ventricular systolic and diastolic dysfunction was prevalent despite successful ART [5]. Moreover, the levels of inflammation were a strong predictor of systolic dysfunction [5]. Contradictory findings exist on how ART modifies the conventional biomarkers of inflammation [6], and dyslipidaemia [6–8]. These inconsistent findings further complicate efforts of delineating the precise mechanisms, which link the interplay between the immune continuum and CVDs in PLWHIV on ART. To date, reliable findings on ART induced changes on CVD-risk markers remain elusive. Notably, several studies have reported on the pivotal role that platelets play in linking inflammation and CVD.

Incongruent findings on the levels of platelets activation in PLWHIV have been reported in several observational studies [6–9]. However, the impact of ART on circulating platelets remains unclear. Notably, antiretroviral drugs have been shown to directly activate platelets, in vitro [10], while individual observational studies have reported on conflicting findings of attenuated [6, 11] and persistently elevated levels of platelet activation following initiation of ART [7–9, 12, 13]. However, the importance and clinical relevance of evaluating platelet activation in the thrombotic-risk stratification of PLWHIV on ART is confounded by variance in the study setting, duration of ART exposure and differences in the methods of enumerating activated peripheral blood platelets.

This systematic review and meta-analysis, therefore, aimed at providing a comprehensive synthesis of studies reporting on the levels of platelets activation in adult patients living with HIV. The primary objective of this study was to determine whether the levels of platelet activation are elevated in treatment naïve patients with HIV, while the secondary objective was to determine whether the levels of platelet activation are attenuated, following successful ART.

## Methods

This systematic review and meta-analysis was prepared and conducted following the preferred reporting items for systematic reviews and meta-analysis (PRISMA) statement [14]. The review was registered on the prospective register of a systematic reviews registry (PROSPERO) (registration number: CRD42017062393), and the study protocol was published [15]. A comprehensive and systematic search of published studies was conducted to address the following research questions:

1. Are platelets activated in patients living with HIV?
2. Does successful ART attenuate the levels of activated platelets in patients with HIV?

### Sources of evidence and search strategy

A comprehensive search was performed on the MEDLINE, Academic search complete, CINAHL with full-text, Health Source: Nursing/Academic edition, and APA Psycinfo databases using the EBSCOHOST search engine. We further searched the Cochrane Central Register of Clinical Trials (Wiley interface), the metaregister (www.controlled-trials.com/mrct/), ISI web of science, and the Global Index of Medicus. Two reviewers (BBN and PVD) independently searched the electronic databases from inception through the 30th of November 2019 using the following search terms: “Platelets” OR “Thrombocytes” OR “Platelet P-selectin” OR “Platelet CD40L” OR “Platelet monocyte aggregates” OR “Platelet leukocytes aggregates” AND “HIV” OR “HIV-1” AND “Antiretroviral therapy”. We further scanned the reference list of selected studies for additional relevant studies. No language restrictions were applied.

### Study selection

The screening of the titles and abstracts of all studies reporting on platelet activation in HIV-infected patients on antiretroviral therapy was independently conducted by three reviewers (BBN, TMN and PVD). Reviews, case reports and pre-clinical studies were excluded. Abstracts from conference proceedings as well as grey literature were also excluded, due to the reported inconsistencies between findings reported in conference abstracts and full publications [16]. The selected studies were also scanned for potential duplicate data publications that may arise from overlapping cohorts reported in different publications. Studies were selected and included in the meta-analysis based on the availability of study-level data required for the effect size estimation.

### Data extraction and management

Two independent reviewers (BBN and PVD) extracted detailed study information and characteristics using a predefined standardised data extraction form. The extracted data items included details of the author, year of publication, original language, sample size, years of follow-up, effect measures reported, gender ratio, levels of coagulation markers, platelet counts, levels of inflammatory markers, mean CD4 counts, HIV-1 RNA levels and HAART regimen. We further extracted the methods used to determine HIV-1 RNA, platelet function and the methods of platelet separation. The extracted data was cross-checked, and inconsistencies were resolved by discussion or referred to a third reviewer (MZ) for arbitration.

### Outcomes

The primary outcomes assessed included levels of platelet activation: reported as the standardised mean difference in soluble platelet P-selectin (sCD62P) or P-selectin (CD62P) levels and soluble CD40L (sCD40L).

### Assessment of risk of bias in included studies

The quality of the included studies was independently assessed by two reviewers (ZM and VM) using the modified Downs and Black checklist which is suitable for evaluating both RCTs and non-controlled trials [17]. The checklist assesses four domains which include (I) reporting bias, (II) external validity, (III) internal validity and (IV) selection bias. The overall scores were graded as excellent (26–28), good (20–25), fair (15–19) and poor (≤ 14). These corresponded to those previously reported [18]. Reviewer scores were cross-checked by two reviewers (TMN and VM) and discrepancies were resolved either through discussions or arbitration by BBN.

### Data synthesis and statistical analysis

Descriptive data items on platelet function in adult HIV-infected patients were summarised in studies which inadequate data for a meta-analysis. The Kappa Cohen’s Kappa (*ĸ*) was used to assess the inter-rater agreement of the reviewer’s quality agreements and scored as no agreement (*ĸ* ≤ 0.00); none to slight (*ĸ* = 0.01–0.20); fair (*ĸ* = 0.41–6.0); substantial (*ĸ* = 0.61–0.80) and almost perfect (*ĸ* = 0.81–1.00).

### Sensitivity analysis and publication bias

Heterogeneity was assessed using the *I*^2^ statistic and a value of 50% was considered as substantial heterogeneity [19], with a *p* value for heterogeneity testing set at < 0.05. Continuous outcomes were pooled as standardised mean differences (SMD), and due to the few number of studies included in the meta-analysis, the Hedges’ *g* statistic (*g*) was used to correct for small study bias. In studies that reported medians and interquartile ranges, we estimated the mean as previously described [20]. Publication bias was assessed using funnel plot analysis, the Egger’s regression test. A *p* value of < 0.05 represented significant levels of publication bias and the trim and fill method was used to identify and compute an adjusted effect size. The sensitivity analysis was performed to test the unexplained sources heterogeneity and robustness of the reported effect estimates. We assessed the effect of each study on the overall standardised mean difference (SMD), by performing a repeated meta-analysis following the omission of a single-study at a time. All analysis was performed using STATA 16.0 (StataCorp LP, TX, USA). All *p* values for associations were two-sided and the value of < 0.05 was considered as significant.

### Patient and public involvement

It was not appropriate or possible to involve patients or the public in the design, or conduct, or reporting, or dissemination plans of our research.

## Results

### Study selection

**Fig. 1:**
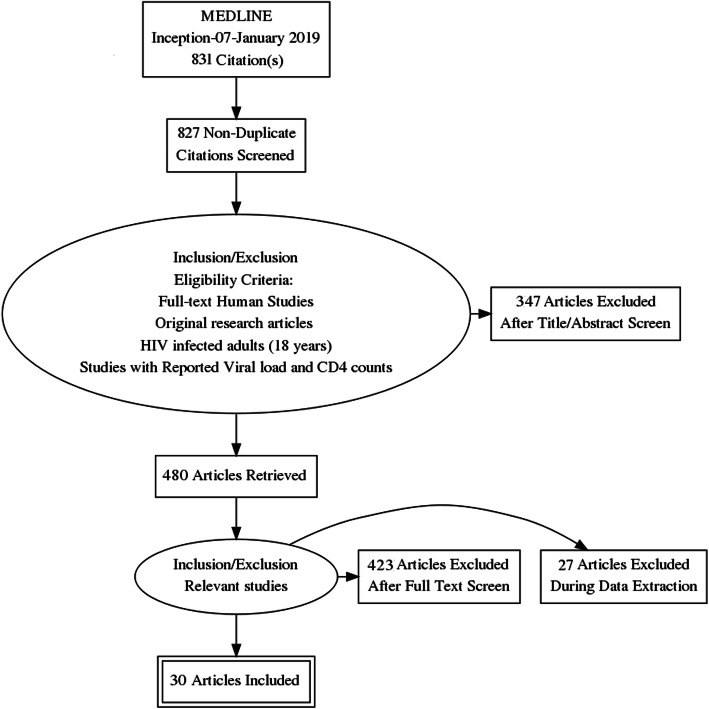
Flow diagram of the study selection process

We identified 831 citations through the electronic database search (390 in MEDLINE, 284 in Academic Search Complete, 73 in CINAHL, 72 in Health Source: Nursing/Academic edition, and 8 in APA Psycinfo and 4 through other sources). After the initial duplicate removal and screening of abstracts, 827 studies were deemed irrelevant and excluded (Fig. 1). The full texts of the remaining 323 studies were assessed for eligibility, and only 30 studies fulfilled the pre-specified inclusion criteria. The 30 studies were all published in English. These included 2 randomised control trial [21, 22] and 28 non-randomised studies [6–9, 11, 12, 22–44].

### Characteristics and quality of eligible studies

The characteristics of the included studies are shown in Table 1. In total, the included studies comprised of 2325 participants. The risk of bias for all included studies was evaluated by two independent reviewers (TMN and VM) using the Blacks and Downs checklist [17]. (Additional file 2: Table S1). The overall inter-rater reliability was assessed using Cohen’s Kappa. The levels of agreement for the various domains were scored as substantial (*ĸ* = 0.81) for the reporting bias and external validity, substantial (*ĸ* = 0.75) for internal validity and almost perfect (*ĸ* = 0.81) for selection bias. Majority of the included studies were rated as poor (76%, *n* = 27) while only 20% of the included studies were rated as fair (Additional file 2: Table S1).

**Table 1:** Characteristics of included studies (*n* = 30)

Author	Year	Study design	Country	Assay	No.	No. HIV+	ART-treated	Gender no. (%Male)	CD4 count	Log HIV-1 RNA levels (copies/ml)	Platelet counts (× 10^**3**^/μL)	Quality assessment *Reporting bias (10)/external validity (3)/internal validity (7)/selection bias (6)*
Aukrust	1997	CS	Norway	EIA	73	53	41	75.47	*NR*	3.3 ± 2.6	*NR*	6/0/2/0
Baker	2012	RCT	USA	ELISA	248	248	134	75.0	1055.33 ± 23.5	5.01 ± 4.05	*NR*	8/0/4/1
Bordoni	2020	PCO	Italy	Luminex	50	43	43	*NR*	1351.5 ± 83	6.34 ± 5.51	*NR*	6/0/4/1
Corrales-medina	2010	CS	USA	FC	55	28	28	*NR*	*NR*	< 1.7	*NR*	8/1/3/1
Damien	2013	CS	France	ELISA	111	71	41	34 (83)	1427.5 ± 77.5^a^	< 1.7	540 ± 18.25^a^	7/1/4/2
Davison	2013	CS	USA	ELISA	35	23	23		434 ± 303.4	< 2.6	*NR*	5/0/4/0
Larranaga	2006	CS	Argentina	ELISA	85	85	35	56 (66)	967.06	5.33 ± 5.42	*NR*	6/0/3/0
De Luca	2000	CS	Italy	ELISA	67	57	37		45.5 ± 4.75	5.8 ± 5	*NR*	6/0/4/1
Donhauser	2012	PCO	Switzerland	ELISA	130	114	62	48 (77.4)	1098.5 ± 88.5	5.8 ± 1.3	*NR*	6/1/3/0
Francisci	2009	RCO	Italy	CBA	84	56	56	43 (76.8)	206.4 ± 196.6	4.96 ± 0.5^a^	*NR*	10/2/5/3
Guzmán-Fulgencio	2011	CS	Spain	CBA	96	73	73	58 (79.5)	1576.67 ± 78.2	*UD*	*NR*	8/0/3/2
Jenabian	2014	CS	Canada	Luminex	55	37	41	41 (74.5)	533.17 ± 269.2	< 1.6^c^	265.64 ± 103	8/0/3/0
Kalayjian	2009	CC	USA	ELISA	152	152	41	98 (88)	257.1 ± 54.5	2.83 ± 2.25	*NR*	10/0/4/2
Kasang	2012	CS	Germany	ELISA	40	30	10	33 (83)	1225.62 ± 87.7	2.0 ± 1.90^d^	*NR*	7/0/3/0
Kieballa	2015	CS	USA	FC	98	37	37	29 (78)	556 ± 248	2.16 ± 2.20	*NR*	7//1/3/0
LandrØ	2011	PCO	Norway	ELISA	84	60	30	45 (75)	151 ± 21	2.99 ± 2.4	*NR*	6/1/4/0
Li	2015	RCO	USA	Luminex	163	122	80	55 (69)	1644.4 ± 365.8	1.50 ± 1.71	*NR*	8/0/3/0
Mesquita	2018	CS	Brazil	FC	66	30	30	14 (46.6)	1383 ± 151^a^	< 1.7	458.75 ± 17.5	7/1/3/1
O’Brien	2013	PCO	USA	LTA, ELISA	69	25	25	19 (76)	1620 ± 235.7 ^a^	< 1.7	501.5 ± 19.25	8/1/3/3
O’Halloran	2015	PCO	Ireland	ECL	40	25	25	15 (60)	827 ± 49.5 ^a^	4.39 ± 0.13^a^	662 ± 20	8/1/4/2
Pastori	2015	CS	Italy	ELISA	70	50	36	25 (69.4)	786.9 ± 354.6	< 1.7	191.8 ± 12.9	8/3/3/3
Quiros-Roldan	2020	RCO	Italy	ELISA	36	36	36	30 (83.3)	429.5 ± 249.2	4.39 ± 4.51	*NR*	8/0/4/2
Rōnsholt	2013	CS	Denmark	Luminex	86	70	70	64 (91)	630	< 1.4^b^	531.9 ± 34.40	7/1/4/3
Satchell	2010	CC	Ireland	LTA	20	20	16	13 (65)	773.5 ± 69.25^a^	3.44 ±	536.5 ± 26.75	7/1/3/0
Singh	2014	CC	USA	FC	17	8	8	*NR*	*NR*	*NR*	*NR*	7/0/3/0
Sipas	2002	CS	Greece	ELISA	94	77	77	67 (85)	335 ± 248	5.03 ± 4.2	*NR*	6/0/3/0
Tunguputri	2014	CS	The Netherlands	FC	110	80	80	64 (80)	683.5 ± 248.6	≤ 1.6	215.8 ± 44	7/1/4/2
von Hentig	2008	CC	Germany	FC	18	18	18	16 (88)	145 ± 112	5.08 ± 0.64	*NR*	7/1/3/2
Wolf	2012	Cohort	Switzerland	ELISA	61	40	40	39 (97.5)	764 ± 155.2 ^a^	2.3 ± 1.95	202 ± 89	8/2/4/3
Wooten	2013	RCT	USA	ELISA	129	107	107	98 (91.6)	504.3 ± 302.05	2.31 ± 3.70	*NR*	6/0/3/1

*NR* not reported, *UD* undetectable, *CS* cross-sectional, *RCO* retrospective cohort, *PCO* prospective cohort, *CC* case-control

^a^The mean and standard deviation were computed using the method described by Hozo et al. [20]

^b^Plasma HIV RNA levels were below 40 copies/mL in a median of 86.7% of measurements during the duration of treatment

^c^In successful ART and elite controllers, while the mean (SD) CD4 count was 4.6 ± 0.8

^d^Undetectable in 90% of HIV-infected patients on treatment (*n* = 27)

### Geographic mapping of the included studies

The included studies comprised of a total of 18 studies conducted in Europe [7, 8, 11, 12, 25–28, 33, 34, 36, 38–41, 43, 45] and 12 studies from the Americas [6, 9, 21, 22, 24, 31, 32, 37, 46]. Studies from Italy [8, 26, 33, 34, 43], Ireland [11, 36] and USA [6, 9, 21, 22, 24, 30, 32, 46] make the highest contribution (Supplementary Figure 1). The bibliometric analysis based on co-authorship links was performed to assess the potential duplication of published datasets.

### Data synthesis and publication bias analysis

In total, the included studies (*n* = 30) comprised of 1725 HIV-infected patients and 600 HIV-negative individuals. Most of the patients with HIV (71%, *n* = 1230) were on various antiretroviral drugs (Table 2). The included studies also reported on various effect measures of platelet activation which included soluble P-selectin (sCD62P), soluble glycoprotein VI (sCD36), soluble CD40L and chemokine ligand 5 (RANTES) (Table 3). In nine of the studies, the included cohort of patients with HIV were virologically suppressed [6, 7, 31–33, 39] with only seven studies reporting on patients with CD4 counts < 500 cells/mm^3^ [8, 12].

**Table 2:** A qualitative summary of the findings on platelet function in antiretroviral-treated individuals in the included studies (*n* = 30)

		Antiretroviral drugs used	Summary of findings on platelet activation and function in HIV-infected patients on antiretroviral therapy		
Author	Year	NRTI	NNRTI	PI	
**Aukrust** [45]	1997	AZT+3TC (26%)	–	IDV (26%)	The serum RANTES levels were elevated in HIV-1 infected patients. In addition, the levels of RANTES directly correlated with CD4 counts and inversely correlated with plasma viral load. Notably, the serum RANTES levels were further increased following the initiation of IDV containing ART.
**Baker** [21]	2012	–	56% of patients NNRTI	29.1% of patients on PIs	The initiation of ART showed no significant reduction in the plasma levels of sCD62P and sCD40L. In addition, the basal sCD40L and sCD62P showed a direct correlation and inversely correlated with platelet counts.
**Bordoni** [43]	2020	All patients (100%) were on NRTIs	4.6% of patients on NNRTIs	72.1% of patients on PIs	The plasma RANTES levels were elevated in patients living with HIV. Moreover, these levels gradually increased despite the initiation of ART.
**Corrales-medina** [6]	2010	3TC (68%) ABC (46%) TDF (43%) FTC (14%) AZT (39%) d4T (14%) DDI (3%)	EFV (36%); NVP (18%)	Lopinavir (18%) NFV (7%) IDV (7%) ATV (7%) FPV (7%)	There were no differences in the levels of CD62P expression in HIV-infected patients on treatment compared to uninfected controls. However, the levels of platelet-derived microparticles were elevated in HIV-infected patients on antiretroviral treatment when compared to uninfected controls. Moreover, the levels of platelet-derived microparticles and activated platelets were similar between patients living with HIV on PI-based therapy or ABC compared to those who were not on PIs or ABC.
**Damien** [7]	2013	None	46% of patients on NNRTIs	54% of patients on PIs (PI regime not reported)	The levels of platelet activation were elevated in HIV-infected patients compared to uninfected controls. Notably, the plasma levels of sCD62P in HIV-infected patients on antiretroviral treatment were 2-fold higher compared to treatment-naïve patients and 3-fold higher, compared to uninfected controls.
**Davidson** [46]	2013	ABC + 3TC (34%)	EFV (37%)	LPV/r (29%)	NNRTI administration was associated with elevated plasma sCD40L. EFV induces the release of sCD40L and also activates the glycogen synthase kinase 3 beta (GSK3β) in platelets.
**De Luca** [26]	2000	65% of patients on NRTIs	–	–	The baseline RANTES levels were compared between treatment-naïve HIV-infected patients, ART-treated and uninfected controls. Notably RANTES were produced at a higher level in the late-stage HIV infected followed by asymptomatic HIV-infected individuals. In addition, plasma RANTES levels were significantly reduced following treatment.
**Donhauser** [27]	2012	*NR*	*NR*	*NR*	The levels of sCD40L were elevated in HIV-infected patients when compared to uninfected controls. Notably, these levels were attenuated by successful antiretroviral therapy and were significantly lower when compared to treatment-naïve HIV-infected patients. However, the levels of sCD40L were not normalised following HAART.
**Francisci** [8]	2009	None	EFV (86%) NVP (14%)	LPV/r (93%) NFV (7%)	The levels of platelet activation were elevated in HIV-infected patients compared to uninfected controls. Notably, only the levels of sCD62P were elevated in patients infected with HIV. While the levels of sCD40L were similar between the HIV and control group. Interestingly, the levels of sCD62P remained persistently elevated post 24 months of antiretroviral therapy.
**Guzmán-Fulgencio** [28]	2011	95.9% of patients on NRTIs	78.5% of patients on NNRTIs	76.7% of patients on PIs	Patients living with HIV had significantly higher levels of sCD40L and sCD62P despite successful HAART. Notably, these showed no significant associations with the risk of cardiovascular events.
**Kalayjian** [24]	2010	d4T DDI 3TC	EFV	NFV LPV/r	The levels of sCD40L remained unchanged following HAART. In addition, higher baseline sCD40L were associated with the incidence of de novo AIDS-defining illness or mortality despite the initiation of HAART.
**Kiebala** [9]	2015	*NR*	*NR*	*NR*	The levels of sCD62P were elevated in HIV-infected patients compared to uninfected controls. These elevated levels persisted even during antiretroviral treatment. In, addition platelet activation is not dependent on IKKB signalling
**Li** [30]	2013	*NR*	*NR*	*NR*	A two-fold increase in sCD40L was reported in treatment-naïve HIV-infected elite controllers when compared to ART-treated or uninfected controls. While the levels of RANTES were lower in elite controllers when compared to treatment-naïve and uninfected controls. In addition, the levels of sCD40L and RANTES showed no correlation with the levels of HIV RNA levels.
**Jenabian** [23]	2014	*NR*	*NR*	*NR*	The levels of sCD40L were elevated chronically infected treatment-naïve HIV-infected patients compared to healthy controls. However, these were normalised following successful ART.
**Kasang** [25]	2012	*NR*	*NR*	*NR*	The levels of sCD40L were lower in HIV-infected patients on HAART compared to treatment naïve patients.
**LandrØ** [12]	2011	*NR*	*NR*	*NR*	Increased levels of sCD62P, sCD40L, RANTES and NAP-2 in HIV-infected patients compared to uninfected controls. These levels were persistently elevated post 24 months of antiretroviral therapy. In addition, the levels of NAP-2 were markedly significantly increased after the initiation of antiretroviral therapy.
**De Larrañaga** [29]	2006	*NR*	*NR*	70%	The levels of sCD62P were elevated in HIV patients on HAART
**Mesquita** [31]	2018	60%	40%	30%	Increased levels of platelet activation, mitochondrial dysfunction and apoptosis in virologically suppressed HIV-infected individuals. Moreover, HIV-infected patients had elevated levels of platelet exhaustion.
**O’Brien** [32]	2013	RAL (16%) ABC (20%)	36% of patients were on NNRTIs	52% of patients were on PIs	The levels of sCD62P were comparable between HIV-infected patients and uninfected controls. Notably, the levels of urinary 11-dehydro-(TX) B2 were elevated in HIV-infected patients compared to uninfected controls. HIV-infected patients who were on antiretroviral therapy and were virally suppressed had elevated levels of activated platelets that were also hyper-reactive. In HIV-infected patients, platelets have a lower activation threshold and display dose-dependent hyper-reactivity. Moreover, Cyclo- oxygenase-1 reactivity remained higher in HIV-infected individuals compared to uninfected controls. Aspirin failed to inhibit arachidonic acid and thromboxane A2 mediated platelet activation.
**O’Halloran** [11]	2015	TDF/FTC	68% of patients were on NNRTIs	None	The levels of sCD62P, sCD40L and sGPVI were elevated in HIV-infected treatment-naïve patients compared to uninfected controls. All markers of platelet activation remained elevated following 3 months of antiretroviral therapy. However, these normalised post 12 months of antiretroviral therapy.
**Pastori** [33]	2015	3TC/AZT or FTC/TDF	64% of patients were on NNRTIs	36% of patients were on PIs	The levels of sCD40L and platelet oxidative stress were increased in HIV-infected patients compared to uninfected controls.
**Quiros-Roldan** [34]	2020	RAL (25%)			The levels of RANTES remained unchanged despite successful ART. Notably, the INSTIs (RAL, EVG and DTG) were associated with a 18–21% increase in RANTES whereas treatment with PI was associated with 32% decrease in serum RANTES levels although these were not statistically significant.
**Rӧnsholt** [35]	2013	NR	NR	NR	The levels of sCD62P were comparable between HIV-infected patients on antiretroviral therapy and uninfected controls.
**Satchell** [36]	2010	ABC (38%) TDF (44%) AZT (19%)	50% of patients were on NNRTIs	None	HIV-infected patients on treatment had decreased reactivity to TRAP, ADP and collagen. In treated HIV-infected patients, a decreased platelet response to TRAP was associated with a lower BMI, total LDL cholesterol and elevated CD8 count. While decreased platelet reactivity to ADP was associated with lower levels of hsCRP. Moreover, an increased platelet response to epinephrine in HIV-infected patients was associated with a lower CD4 count and increased CD8 count. Whereas a history of CVDs was associated with decreased response to epinephrine.
**Singh** [37]	2014	NR	NR	NR	The levels of platelet monocyte aggregates are elevated in patients with HIV and these persist despite successful ART
**Sipsas** [38]	2002	NR	NR	NR	The levels of sCD40L were two-fold higher in HIV-infected patients compared uninfected controls. In addition, these were threefold higher following 8–12 months of HAART.
**Tunguputri** [39]	2014	RAL (31.25%) ABC (6%)	EFV; RPV or NVP ABC 9%	RTV-boosted PI (DRV, ATV or LPV) ABC (3%)	Increased levels of platelet monocyte aggregates in HIV-infected patients persist despite effective ART. Notably, RAL-based regimen lowered platelet hyperactivity and platelet monocyte aggregates. While ABC-treated patients showed a trend of higher platelet reactivity.
**Wolf** [41]	2012	2.5% of patients were on NRTIs	NVP (10%) EFV (10%)	RTV (5%) IDV (5%) SQV (37.5%)	The levels of platelet activation were higher in HIV-infected patients when compared to controls. The levels of sCD62P and sCD40L did not decrease during antiretroviral therapy.
**Von Hentig** [40]	2008	–	–	SQV/LPV/r (22%) SQV/RTV (61%) FPV (11%) FPV/LPV/r (6%)	Short-term 4-week ART treatment with PIs enhanced the levels of CD40L and CD41 activity on platelets. Whereas CD62P levels were comparable after ART.
**Wooten** [22]	2013	NR	NR	NR	The plasma levels of RANTES were significantly elevated in patients with HIV on stable HAART for a duration of 6 months.

*NRTIs* nucleotide reverse-transcriptase inhibitors, *NNRTIs* non-nucleotide reverse-transcriptase inhibitors, *PI* protease inhibitor, *TDF* tenofovir disoproxil fumarate, *TAF* tenofovir alafenamide fumarate, *FTC* emtricitabine, *ABC* abacavir, *AZT* zidovudine, *NFV* nelfinavir, *NR* not reported, *IDV* indinavir, *ATV* atazanavir, *FPV* fosamprenavir, *3TC* lamivudine, *RAL* raltegravir, *d4T* stavudine, *DDI* didanosine, *EFV* efavirenz, *RPV* riplivirine, *NVP* nevirapine, *RTV* ratinovir, *SQV* saquinavir, *DRV* darunavir, *ATZ* atava, *LPV/r* lapinavir/ritonavir, *NFV* nelfinavir, *EVG* elvitegravir, *DTG* dolutegravir

**Table 3:** Reported markers of platelet activation in treatment-naïve and ART-treated HIV-infected patients

Effect measure	No. studies	Levels of platelet activation in PLWHIV	
		Treatment naïve	Post-ART
sCD62P	12	Increased [6–9, 11, 21, 28, 31, 33, 41] similar between PLWHIV and controls [6]	Increased [6–9, 12, 21, 28, 29, 31]; decreased [11, 35, 40]
sCD40L	14	Increased [6–8, 10, 13, 30, 34, 36–38, 43] Similar between PLWHIV and controls [8]	Increased [7, 11, 21, 24, 27, 28, 38, 40, 46]; Increased in NNRTI-treated patients [25, 41]; Decreased in [7, 28, 37–39]
sGPVI	1	Increased [11]	Decreased [11]
RANTES	6	Increased [10, 29–33]	Increased [10, 29, 31, 45]; Decreased [26]
CD62P	3	Increased [9]	Increased [31]; Decreased In RAL-treated patients; Similar between PLWHIV and controls [6]
PMAs	2	Increased [39]	Decreased in RAL compared to NNRTI- and PI-treated PLWHIV [39]

*sCD62P* soluble P-selectin, *sCD40L* soluble CD40 Ligand, *sGPVI* soluble glycoprotein VI, *RANTES* chemokine ligand 5, *CD62P* P-Selectin, *PMAs* platelet monocyte aggregates, *RAL* raltegravir

The Egger’s regression test suggested evidence of publication bias (*p* < 0.001) (Additional file 1: Table S2). In addition, the funnel plot analysis indicated asymmetry as the trim-and-fill method imputed no missing study for the primary platelet activation outcome (Additional file 1: Table S2 and Figure S1). Notably no missing study was imputed (based on the trim and fill method) for comparisons of platelet activation between ART-treated treatment-naïve (Additional file 1: Table S2).

### Platelet activation in HIV-infected compared to uninfected patients

**Fig. 2:**
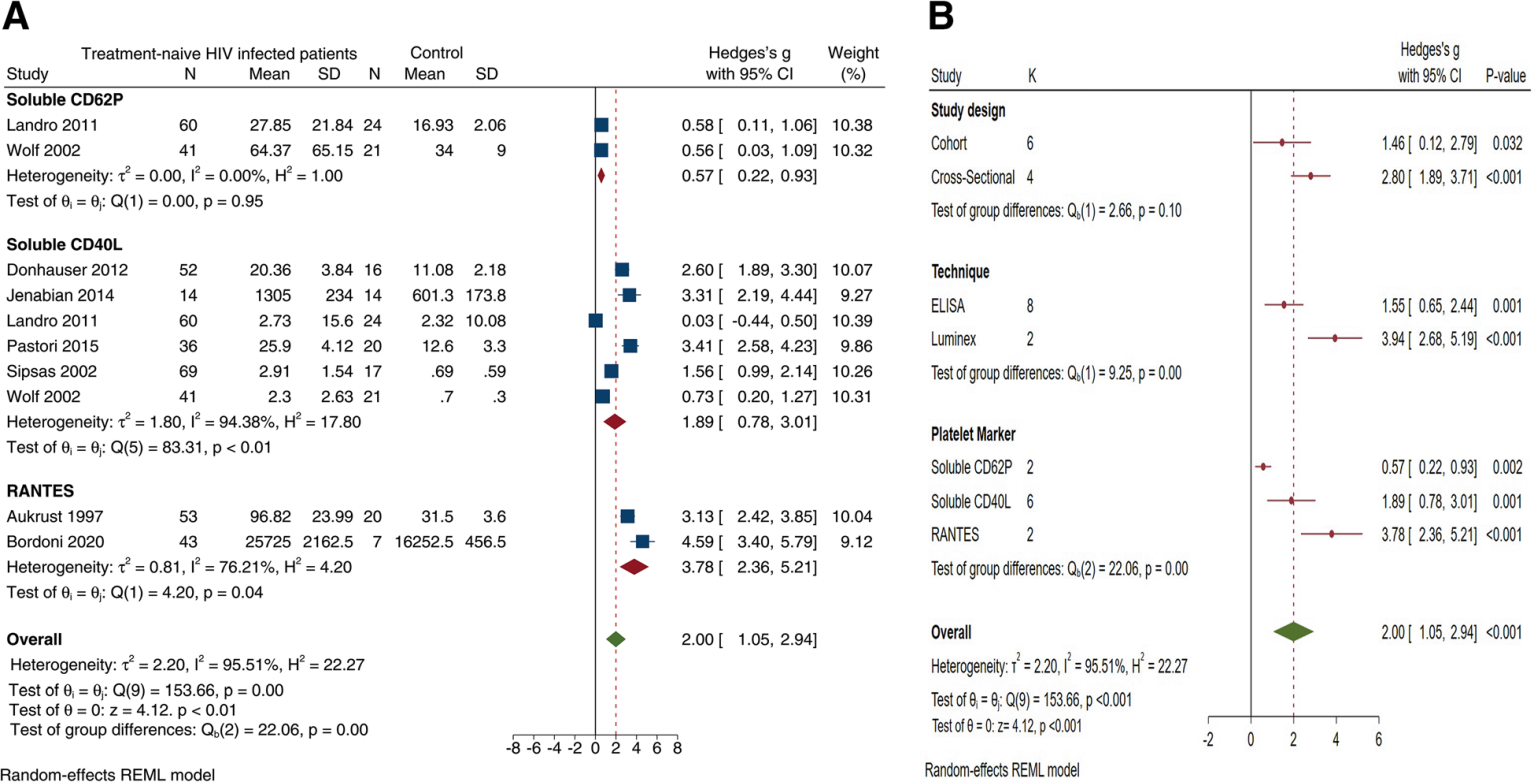
The association between platelet activation and HIV infection. **a** The forest plot shows the pooled effect estimate of platelet activation in treatment-naïve HIV-infected patients compared to uninfected controls. **b** The subgroup effects based on the methodological differences in the included studies

In the majority of the studies (61%, *n* = 17), the levels of platelet activation were elevated in HIV-infected patients compared to uninfected controls. The included studies reported on various surrogate markers of platelet activation; these included markers of alpha granule secretion (RANTES) and markers of platelet adhesion and activation (CD62P, CD40L and platelet-monocyte aggregates) (shown in Table 3). In order to assess whether the varying methods used modified the reported effect estimate, we performed a subgroup analysis based on the type of method used in each study. In our subgroup meta-analysis, the primary outcome of platelet activation in HIV-infected patients was compared to uninfected controls. We observed a quantitative effect based on the methodology used to enumerate the levels of platelet activation, whereby in studies using the Luminex, the largest effect size (Hedges’ *g* 3.94 [95%CI 2.68, 5.19], *p* < 0.001) was compared to ELISA-based technology (Hedges’ *g* 1.55 [95%CI 0.65, 2.44], *p* = 0.001) (Fig. 2b). Notably, both methods reported on similar direction of the effect and significantly increased levels of platelet activation in HIV-infected patients.

In all, elevated levels of the surrogate markers of platelet activation, CD40L, P-selectin (CD62P) and the soluble form (sCD62P and sCD40L), were consistently reported in HIV-infected patients [7–9, 11, 12, 23, 24, 27, 30, 31, 33, 37–39, 41, 43, 45]. While a few studies (17%, *n* = 2) reported on comparable levels of sCD40L [8], RANTES [26], sCD62P and CD62P [6, 47]. However, the random-effects model, which included 8 studies reporting on 409 treatment-naïve HIV-infected patients and 138 uninfected controls, demonstrated an overall increase in the levels of platelet activation in HIV-infected patients compared to uninfected controls (Fig. 2). The overall bias-corrected standardised mean difference (Hedges’ *g*) in the levels of platelet activation in treatment-naïve HIV-infected patients compared to uninfected controls was 2.00 [95%CI 1.05, 2.94]; *z* = 4.12, *p* < 0.001. Notably, the levels of heterogeneity were high (*I*^2^ = 95.91%, *p* < 0.01). Therefore, we performed a subgroup analysis to explore the sources of heterogeneity based on the reported marker of platelet activation. The test for subgroup effects showed a significant subgroup effect (*p* < 0.001) (Fig. 2a). This suggests that the reported pooled effect estimate was influenced by the various markers of platelet activation reported in the included studies.

#### Platelet P-selectin levels in treatment-naïve HIV-infected patients

A total of 16 included studies reported on the levels of P-Selectin (CD62P) in untreated HIV-1-infected patients compared to ART-treated and uninfected controls [6–8, 11, 12, 21, 28, 29, 31, 32, 35, 37, 39–41]. However, only two studies [12, 41] had adequate study-level data and were included in the subgroup meta-analysis. The pooled effect estimate included 146 participants comprising 101 HIV-infected patients and 45 uninfected controls. Interestingly, the sCD62P levels were elevated in patients with HIV when compared to uninfected controls (Hedges’ *g* 0.57 [95%CI 0.22, 0.93], *p* = 0.002). Moreover, there were low levels of heterogeneity (*I*^2^ = 0%) (Fig. 2).

#### Soluble CD40L levels in treatment-naïve HIV-infected patients

A total of 384 participants were included in the subgroup meta-analysis of 6 studies. This comprised of 272 treatment-naïve HIV-infected patients and 112 uninfected controls. The levels of sCD40L were elevated in treatment-naïve HIV-infected patients compared to controls (Hedges’ *g* 1.89 [95%CI 0.78, 3.01], *p* = 0.001) (Fig. 2b). Notably, the levels of heterogeneity were high amongst the included studies (*I*^2^ = 94.38%). We, therefore, explored other sources of heterogeneity which included differences in the study design, and the methodology used to measure the levels of platelet activation (Fig. 2b).

**Fig. 3:**
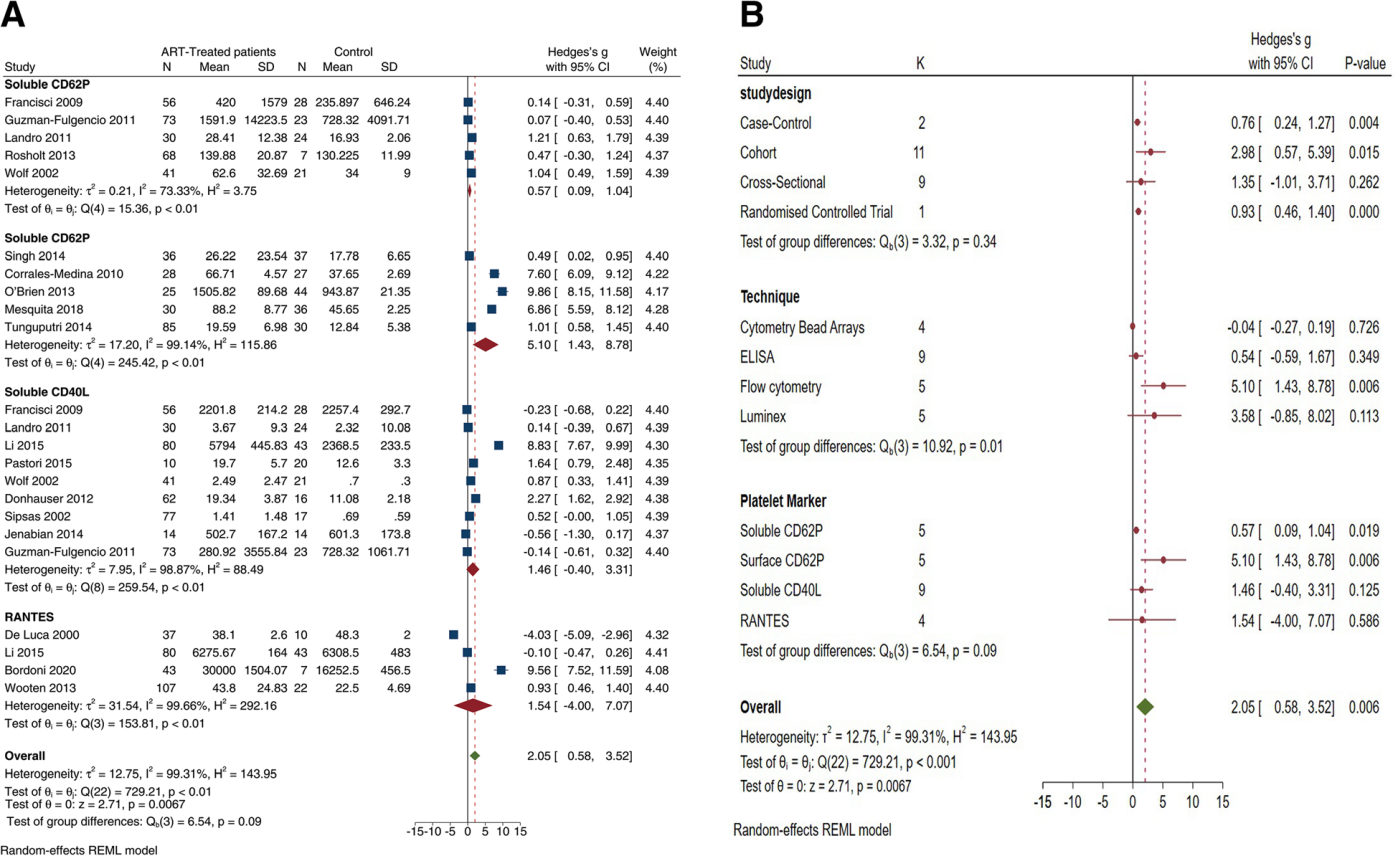
Platelet activation in ART treated compared to uninfected individuals. The forest plot (**a**) shows the pooled effect estimates of platelet activation in treated compared to uninfected patients. **b** The subgroup interactions based on the study design, method used to determine the levels of platelet activation and the reported platelet-associated marker

The test for group differences showed a significant interaction effect based on the reported technique or methodology used (*p* < 0.01) and reported marker of platelet activation (*p* < 0.001) (Fig. 3b). Notably, there were no significant between-study differences on the reported pooled estimates based on the varying study designs (*p* = 0.10).

#### RANTES levels in treatment-naïve HIV-infected patients

A total of 123 participants from 2 studies [43, 45] were included in this subgroup analysis (Fig. 2). In all, this included 96 treatment-naïve HIV-infected patients and 27 uninfected controls. The pooled estimate showed significantly increased levels of RANTES in treatment-naïve HIV-infected patients compared to uninfected controls (Hedges’ *g* 3.78 [95%CI 2.36, 5.21], *p* = < 0.001) (Fig. 2b). However, the levels of heterogeneity were high amongst the studies included in this subgroup (*I*^2^ = 76.21%).

### Platelet activation in HIV-infected patients on antiretroviral therapy compared to treatment-naïve HIV-infected patients

Elevated levels of platelet activation, exhaustion and apoptosis persist even during successful ART [6–9, 11, 12, 21, 22, 24, 27–29, 31–41, 43, 45–47]. The use of NNRTIs was associated with elevated sCD40L levels [41], while others reported that ART did not affect the elevated levels of sCD40L, RANTES and urinary 11-dehydrothromboxane B2 levels [7, 21, 24, 34, 47]. In addition, plasma sCD62P and sCD40L levels were 2–3-fold higher in treated HIV-infected patients compared with treatment-naïve HIV-infected patients [7, 38]. Congruently, persistently activated elevated levels of platelet reactivity have been reported following 6 to 24 months of initiating treatment [7, 8, 22, 38–41, 47]. In contrast, three studies reported on the normalisation of platelet activation levels following 12 months of ART [11, 23, 26]. Moreover, ART-treated patients had higher levels of spontaneous platelet aggregation in response to submaximal concentrations of various endogenous platelet agonists [47].

**Fig. 4:**
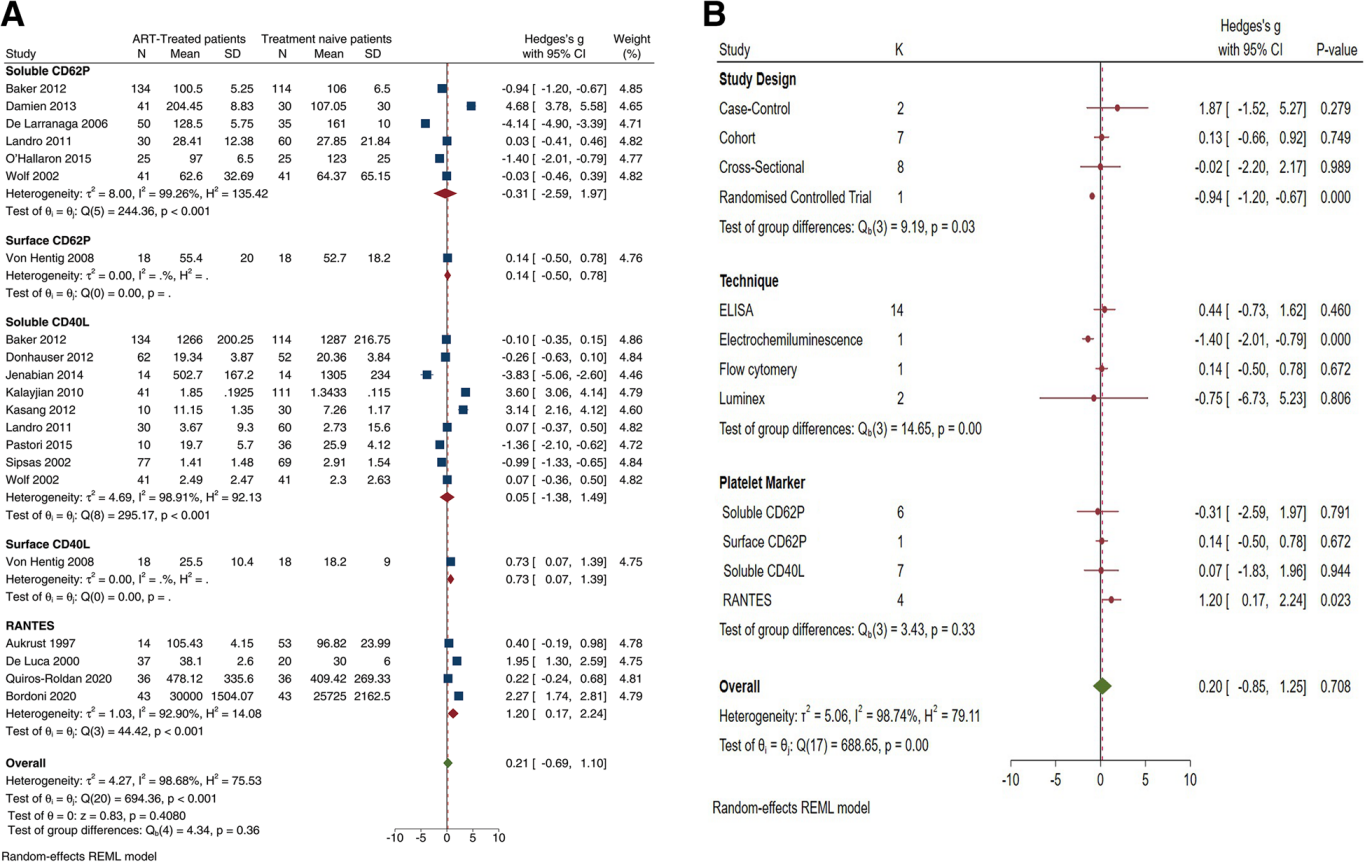
The effects of antiretroviral therapy on the level of platelet activation in HIV-infected patients. The forest plot (**a**) shows the pooled effect estimate of platelet activation levels in HIV-infected patients on antiretroviral therapy (ART) compared to treatment-naïve patients. **b** The subgroup meta-analysis based on the study design, method used to determine the levels of platelet activation and the reported platelet-associated marker

Only 18 studies had adequate study-level data and were included in the meta-analysis [6–8, 11, 12, 22, 23, 26–28, 30, 32, 33, 35, 37–41, 43, 45]. The pooled estimates showed elevated levels of platelet activation in ART-treated patients compared to controls (Hedges’ *g* 2.05 [95%CI 0.58, 3.52]; *z* = 2.71, *p* = 0.0067) (Fig. 3), and these elevated levels were comparable between ART-treated patients with HIV and treatment-naïve patients (Hedges’ *g* 0.21 [95%CI − 0.69, 1.10], z = 0.83 *p* = 0.4080) (Fig. 4). However, these levels of heterogeneity were substantial (*I*^2^ = 98.68%). To explore the sources of unexplained heterogeneity, we then conducted a subgroup analysis based on the reported effect measure of platelet activation within the included studies (Fig. 3a).

In all, the levels of both surface CD62P (Hedges’ *g* 5.10 [95%CI 1.45, 8.78], *p* = 0.006) and sCD62P (Hedges’ *g* 0.57 [95%CI 0.09, 1.04], *p* = 0.019) remained significantly elevated in patients with HIV on ART when compared to uninfected controls (Fig. 3). We further conducted a subgroup meta-analysis based on the study design, the technique used and reported marker of platelet activation (Fig. 3b). The test for subgroup differences demonstrated a statistically significant interaction effect, in the subgroup based on the technique (*p* < 0.01) and marker used to enumerate activated platelets (*p* < 0.09).

## Discussion

To date, this is the first systematic review and meta-analysis of studies reporting on platelet activation in adult patients living with HIV. We retrieved and analysed 30 studies that reported on platelet activation in adult PLWHIV on ART. Several of these studies (50%, *n* = 15) reported on data obtained from serum or plasma samples using enzyme-linked immunosorbent assays. While the methods used to measure platelet activation included the detection of surface markers of platelet activation using flow cytometry (23%, *n* = 7), Luminex technology (10%, *n* = 3) and immunoassay-based methods (50%, *n* = 15). Platelet function was only evaluated in a few studies (1.66%, *n* = 2) using light transmission aggregometry. Notably, the subgroup meta-analysis showed that methodological variance may influence the reported levels of platelet activation, with studies employing the Luminex technology showing a larger pooled estimate when compared to ELISA-based methods.

In all, several studies reported on elevated levels of soluble, CD62P (sCD62P) and CD40L (sCD40L), in PLWHIV compared to uninfected controls (Table 3). This supports the finding of increased levels of platelet activation in adult HIV-infected patients [7, 8, 11, 12, 33, 41]. Moreover, in follow-up studies, these levels were shown to persist following 3 to 24 months of successful ART [8, 12, 22, 38, 47]. Although ART attenuated the levels of platelet activation in patients who were not receiving protease inhibitors (PIs) [11], in those patients on PI-based therapy, the levels of platelet activation persisted despite successful ART [21, 24, 28, 29, 39, 41, 43, 45–47]. Moreover, the levels of platelet activation were 2-fold higher in patients on PI-based therapy compared to treatment naïve patients with HIV [7, 30, 38]. Notably, different measures of platelet activation were reported in the included studies (Table 3); the vast majority of the studies report on congruent findings of elevated levels of platelet activation despite achieving viral suppression, although a few of the included observational studies reported on discordant findings, on the levels of CD62P and CD40L in ART-treated patients living with HIV [7, 8].

To our knowledge, this is the first pooled analysis of platelet activation levels reported in patients living with HIV. The primary objective of the planned meta-analysis was to determine the association between the levels of platelet activation in HIV-infected treatment-naïve patients. While the secondary objective of this analysis of pooled data was to assess whether effective ART attenuates the levels of platelet activation in patients living with HIV. In all, the levels of platelet activation were elevated in adult patients living with HIV when compared to uninfected controls (Fig. 2). Notably, this supports the previously reported findings of elevated levels of platelet activation in HIV-infected patients [6, 13, 36, 48]. The current study adds value in highlighting that sCD40L levels are increased at greater magnitude when compared to levels of sCD62P in treatment-naïve HIV-infected patients. These findings further highlight the potential benefit of using sCD40L and sCD62P in thrombotic-risk profiling of PLWHIV on ART, as sCD62P is associated with platelet and vascular activation.

The present meta-analysis of patients with HIV on ART and treatment-naïve infected patients suggests that ART had no effect on the levels of platelet activation, although the RANTES, sCD40L and surface CD40L levels were comparable following successful ART (Fig. 3b). In contrast, the sC62P and surface CD62P levels remained significantly increased following effective ART (Fig. 3b). Differences in the treatment regimens may account for the incongruent effect estimates. In fact, all studies reporting on patients receiving PI-based ART showed elevated levels of platelet activation despite successful viral suppression [6–8, 39, 47].

The strengths of this review include the comprehensive search and data extraction which was independently performed by two reviewers. The inter-rater agreement was high for most of the risk of bias. Although the included studies showed high levels of heterogeneity, the potential risk of bias of these studies was scored as fair. The presented cumulative evidence is limited by the lack of adequate randomised control trials and reporting of ART regimen used. Due to the lack of adequate number of RCTs addressing the changes in platelet activation following ART, we considered and included observational studies alongside RCTs in the meta-analysis [49]. The included studies were scored low in the external validity, thus limiting the generalisability of these findings. Lastly, the pooled effect estimates on the levels of platelet activation were derived from studies conducted mainly in the Americas and countries in Europe. Caution should be taken in extrapolating these findings into a different geographic setting.

## Conclusion

This meta-analysis provides evidence that the levels of soluble CD62P and CD40L are elevated in HIV-infected patients and that sCD62P levels persist despite successful therapy. Overall, we report on elevated levels of platelet activation in adult PLWHIV which persist despite successful ART.

## Supplementary information

**Additional file 1: Table S1.** Search strategy used on the EBSCOHOST search engine. **Table S2.** Publication bias analysis. **Figure S1.** Analysis of publication bias. The funnel plots were visually inspected for publication bias in the included studies.

**Additional file 2: Table S2.** Risk of bias analysis.

## Data Availability

The authors confirm that the data supporting the findings of this study are available within the article and its additional files.
