# Supplementary material for: Platelet activation in adult HIV-infected patients on antiretroviral therapy: a systematic review and meta-analysis
Source: BMC Med. 2020 Nov 18;18:357. doi: 10.1186/s12916-020-01801-9 (PMC7672943; doi:10.1186/s12916-020-01801-9)
Supplement: Supplementary file 1 — Additional file 1: Table S1. Search strategy used on the EBSCOHOST search engine. Table S2. Publication bias analysis. Figure S1. Analysis of publication bias. The funnel plots were visually inspected for publication bias in the included studies. [file 12916_2020_1801_MOESM1_ESM.pdf]

Online supplementary data

Table S1.Search strategy used on EBSCOHOST (Search ran on the 23 June 2020)

**Databases searched (#Hits)**	**MEDLINE (n=390); Academic Search Complete (n=284); CINAHL with Full text(n=73); APA Psycinfo (n=8)**
**Search Strategy used**	**Concept#1 AND Concept#2 AND Concept#3**
Concept#1	(Platelets[Text Word]) OR (Platelets[MeSH Terms]))
Concept#2	HIV[Text Word])) AND (Human immunodeficiency virus[Text Word])) OR (Human immunodeficiency virus[MeSH Terms])
Concept#3	(Antiretroviral therapy[Text Word])) OR (Anti-retroviral therapy[Text Word])) OR (agents, antiretroviral[MeSH Terms])) OR (highly active antiretroviral therapy[MeSH Terms])

Table S2. Publication bias analysis

Outcome	No. missing studies	Model	Coef. (95%CI)	Z- Value	P-value
PLT activation in ART naïve	0	RE	12.80(10.31-15.28)	11.34	<0.001
PLT activation in ART-treated	0	RE	8.63(4.15-13.10)	3.98	0.006
PLT activation in ART naïve vs. Treated	0	RE	2.29 (11.83-20.81)	7.13	0.001

PLT: Platelet, **RE:** Random effects

A

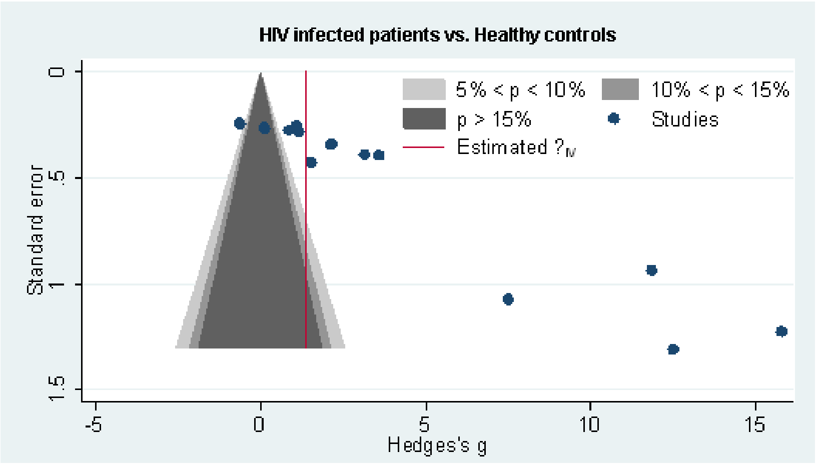

B

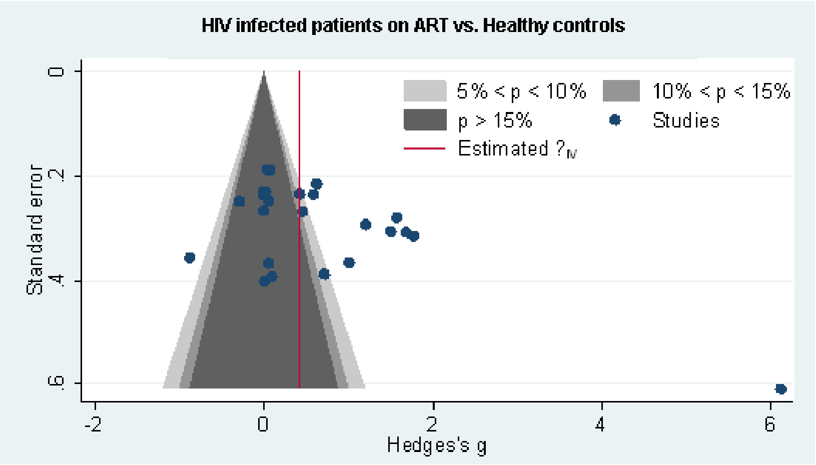

C

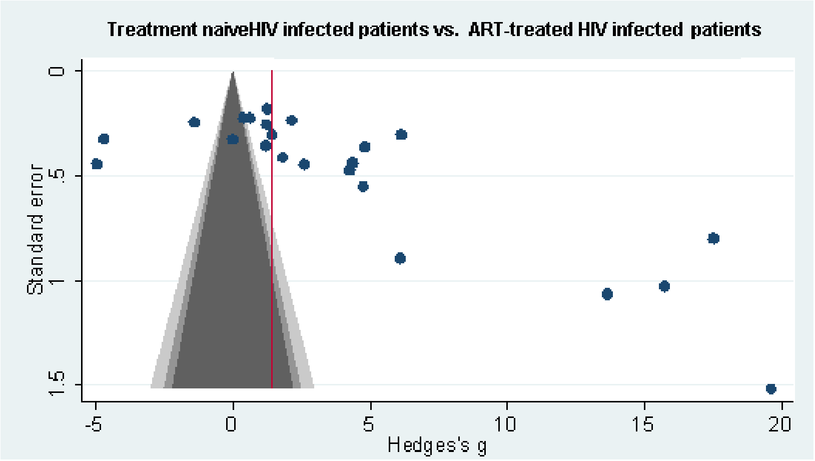

Figure S1.Analysis of publication bias. The funnel plots were visually inspected for publication bias in the included studies.
